# Prevalence of unprotected anal intercourse in men who have sex with men recruited online versus offline: a meta-analysis

**DOI:** 10.1186/1471-2458-14-508

**Published:** 2014-05-26

**Authors:** Zhongrong Yang, Sichao Zhang, Zhengquan Dong, Meihua Jin, Jiankang Han

**Affiliations:** 1 Huzhou Center for Disease Control and Prevention, Huzhou 313000, Zhejiang Province, China

**Keywords:** Epidemiology, HIV, Men who have sex with men, AIDS

## Abstract

**Background:**

Men who have sex with men (MSM) are a high risk population for human immunodeficiency virus (HIV) infection. Our study aims to find whether MSM who were recruited online had a higher prevalence of self-reported unprotected anal intercourse (UAI) than those who were recruited offline.

**Methods:**

A meta-analysis was conducted from the results of published studies. The analysis was stratified by the participants’ geographic location, the sample size and the date of the last reported UAI.

**Results:**

Based on fourteen studies, MSM who were recruited online (online-based group) reported that 33.9% (5,961/17,580) of them had UAI versus 24.9% (2,700/10,853) of MSM who were recruited offline (offline-based group). The results showed that it is more likely for an online-based MSM group to have UAI with male partners than an offline-based MSM group [odds ratio (*OR)* = 1.35, 95% *CI* = 1.13-1.62, *P* < 0.01]. The subgroup analysis results also showed that the prevalence of UAI was higher in the European subsample (*OR* = 1.38, 95% *CI* = 1.17-1.63, *P* < 0.01) and in sample sizes of more than 500 individuals (*OR* = 1.32, 95% *CI* = 1.09-1.61, *P* < 0.01) in the online group compared to the offline group. The prevalence of UAI was also significantly higher when the time of the last UAI was during the last 3 or more months (*OR* = 1.40, 95% *CI* = 1.13-1.74, *P* < 0.05) in the online group compared to the offline group. A sensitivity analysis was used to test the reliability of the results, and it reported that the results remained unchanged and had the same estimates after deleting any one of the included studies.

**Conclusions:**

A substantial percentage of MSM were recruited online, and they were more inclined to engage in UAI than MSM who were recruited offline. Targeted interventions of HIV prevention programs or services are recommended when designing preventive interventions to be delivered via the Internet.

## Background

Currently, the prevalence of human immunodeficiency virus (HIV) among men who have sex with men (MSM) is rapidly increasing worldwide [1-4]. MSM are also at a high risk of infection with sexually transmitted diseases (STDs) [5,6] because of related risky behaviors, such as having multiple partners and engaging in unprotected anal intercourse (UAI) [7]. Many studies showed that MSM seeking male sexual partners (sampled offline or from fixed venues, such as gay bars, bathrooms, or clubs) engage in several risky sexual behaviors, such as UAI, having multiple sex partners and anal sex [8-12]. However, the studies are limited because offline sampling misses MSM who do not go to these venues due to fear of discrimination, and some HIV-positive MSM who are at high risk for transmission of HIV or STDs may not go to these places either.

The recruitment of MSM for studies is a challenge for researchers because no sampling frame exists for MSM and public acknowledgement of membership may also be stigmatized in some cases [13]. The Internet, with its convenience of accessibility to communication, information, entertainment and web-based communities [14], has become a basic tool for MSM who seek sex partners and for arranging liaisons [15]. MSM can find sexual partners through chat rooms or the corresponding social forums online (eg. http://www.gaydar.net/); they also perceive that this method is convenient and cost-effective because it is private, anonymous, safe and convenient in the process of communication. Although many studies have applied offline-based sampling to MSM [16-19], several studies [20-22] sampling MSM who seek male sexual partners via the Internet have shown that such online-based sampling is cost-effective and has lots of advantages.

Some studies have shown that online-based MSM were more likely to report different socio-demographic profiles [1] and risky sexual behaviors, such as self-identified sexual orientation [1,7], UAI [1,23,24] and having multiple sex partners [24,25], compared with offline-based MSM. The online-based sample was significantly younger (Internet sample mean age 33.2 years old, offline 37.6 years old) and was comprised of more bisexual men (Internet sample 20%, offline 5%) than the offline MSM sample [26]. In addition, epidemiological studies performed in European [27,28], American [29] and Asian [1,7] MSMs reported risky sexual behavior (e.g., UAI, having multiple sex partners) and showed an increase in the prevalence of UAI.

It is important to examine the validity of sampling online compared to more established venue-based methods because the individuals recruited by sampling online may be fundamentally different from those recruited offline, with respect to their sexual risks. In the past few decades, several studies [26,28-31] have reported that online-based MSM were more likely to have UAI with male sex partners than offline-based MSM, but the findings are inconsistent and show some conflicting outcomes due to different regions or low statistical power. In our research, we conducted a meta-analysis to assess whether online-based MSM had a higher self-reported UAI prevalence than offline-based MSM.

## Methods

### Search strategy and selection criteria

Studies published before December 2013 that examined the prevalence of UAI among MSM were carefully selected from the following databases: PubMed (1966 to 2013), Springer (1996 to 2013), Cochrane Library (1993 to 2013), Google Scholar (1987 to 2013), China National Knowledge Infrastructure (CNKI, 1979 to 2013) and the Wanfang database (Chinese, 1990 to 2013). The date of the last search was February 8, 2014. The databases were searched using the following key words: “men who have sex with men”, “Internet”, “web”, “online”, “offline”, “venue”, “MSM”, “gay”, “homosexuality”, “risky behavior”, “sexual behavior” or “anal intercourse”. No language restrictions were carried out for this study. All of the studies that investigated the prevalence of UAI among online-based MSM or offline-based MSM were evaluated carefully. The selection criteria are listed as follows: (1) the reports were full-length, published papers; (2) the studies reported data for UAI among MSM, and the duration of UAI was not limited; and (3) all of the studies recruited MSM both online and offline. We excluded the studies in which the reported UAI data were from MSM recruited either only online or offline and any meeting or conference abstract data.

### Search methods

Two investigators (Yang ZR and Zhang SC) reviewed the abstracts independently to determine whether the studies conformed to the eligibility criteria for this study. Two other investigators (Jin MH and Dong ZQ) reviewed the references in the papers to identify any additional studies. A third investigator (Han JK) carried out additional assessments if discrepancies were generated. In our study, there were no discrepancies.

### Data extraction

The data items included study details (e.g., sample size, year of publication, location of participants), characteristics of participants (e.g., age, proportion of MSM), and different risky sexual behaviors (e.g., never used a condom in the process of anal intercourse with partners in the last year, inconsistent condom use during anal intercourse in the last year, UAI with a male partner in the past three months, etc.). Two investigators (Yang ZR and Dong ZQ) extracted the data independently using the standardized protocol by coding forms, and a third investigator (Jin MH) reviewed the results.

For each study, we recorded the first author’s name, the publication year, the country and geographic location, the definition of UAI (risky sexual behaviors, such as UAI in the last x months, UAI with a casual partner, etc.), the sample size and the prevalence of UAI. UAI was defined as having never used a condom during anal sex in the last year, using condoms inconsistently in the process of anal intercourse in the last year, UAI with a serodiscordant or HIV-unknown male partner, UAI with a male partner in the last three months, UAI in the last two months, UAI with any male partner in the last three months, UAI with a casual partner in the last six months, UAI in the last three months, UAI with a male partner in the last incident of anal sex, any UAI with any partner, or being engaged in any MSM UAI in the last year.

### Meta-analysis methods

This study assessed the comparison of the UAI prevalence between MSM who were recruited online (online-based group) vs. those who were recruited offline (offline-based group). The online-based group’s participants were investigated through the Internet (online), and the respondents of the offline-based group were surveyed in bathhouses, bars, clubs, etc. We examined the association with the prevalence of UAI among MSM between the online-based group and the offline-based group. Then, we merged data from the same geographic location, sample size and the last incidence of UAI by means of subgroup analysis.

The odds ratios (*ORs*) and 95% confidence intervals (95% *CI*) were calculated with respect to each study. We quantified the effect of heterogeneity by means of the formula *I*^2^ = 100% × (*Q* − df)/*Q*[32]. We also estimated the within-study and between-study variation or heterogeneity using Cochran’s *Q*-statistic [33]. The random effect model was then used for the meta-analysis if a significant *Q*-statistic (*P* < 0.10) existed; otherwise, the fixed effect model was used [34].

The overall *OR,* or the pooled estimate of risk, was obtained using the Mantel-Haenszel method in the fixed effect model [35] and using the DerSimonian and Laid method in the random effect model [36]. The pooled *OR* in the meta-analysis was calculated by weighting the individual *OR*s using the inverse of their variance [34]. The significance of the pooled *OR* was determined using a Z-test [37]. To test the reliability of the results, we also performed a sensitivity analysis after deleting any one of the included studies.

### Evaluation of publication bias

We measured the asymmetry of the funnel plot by using Egger’s linear regression [38], which assessed funnel plot asymmetry using the natural logarithm scale of the *OR*[34]. The intercept α provides a measure of asymmetry: the larger its deviation from zero, the more pronounced the asymmetry [38].

The meta-analysis was conducted with Review Manager 5.1 software (Cochrane Collaboration, http://tech.cochrane.org/revman) and the STATA software package v.11.0 (Stata Corporation, College Station, TX, USA). All the *P* values were two-sided. Differences were considered statistically significant if the *P* value was less than 0.05.

## Results

### Characteristics of eligible studies

**Figure 1:**
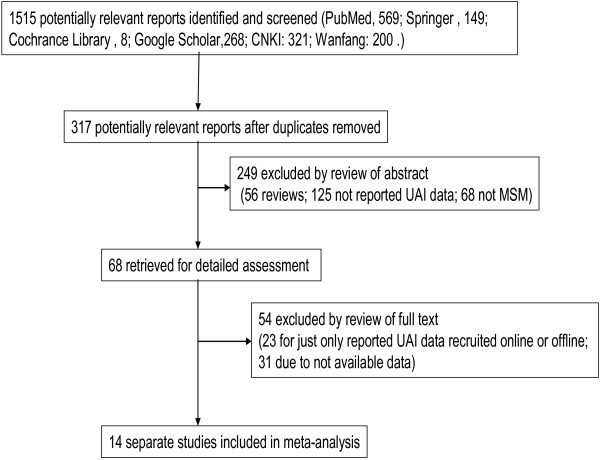
Flow chart of study enrollment for this meta-analysis.

There were 1515 reports or literature pieces related to the search terms (PubMed: 569; Springer: 149; Cochrane Library: 8; Google Scholar: 268; CNKI: 321; Wanfang: 200). The flow chart of study enrollment for this meta-analysis is shown in Figure 1. A total of 317 studies were potentially relevant after duplicate or unrelated studies were excluded. During the abstract screening procedure, 249 papers were removed (56 were review articles; 125 had no data on UAI; 68 did not include MSM). A total of 68 articles were retained for full text review, and 54 papers (23 due to reporting UAI data from MSM recruited either only online or offline; 31 due to unavailability of data) were removed after full text review.

A total of 14 studies published between 2000 and 2012 were included in this meta-analysis. The characteristics of the selected studies are listed in Table 1. There were 28,433 participants (online group 17,580; offline group 10,853) in this meta-analysis. Six of the studies were carried out in Europe [23,26-28,30,31], three in Asia [1,7,39] and five in America [29,40-43]. The prevalence of UAI varied between 9.8% and 59.9% in the online-based group and between 7.5% and 64.9% in the offline-based group. We merged the research data according to the same geographic location, sample size and time of last UAI; we also analyzed these subgroups independently.

**Table 1:** Characteristics of studies included in the meta-analysis

**Study**	**Year**	**Country**	**The definition of UAI**	**Sample size**	**Prevalence of UAI among MSM recruited online**	**Prevalence of UAI among MSM recruited offline**
Ross et al. [30]	2000	Sweden	Never used condom during AI with casual partners in the last year	MSM (overall), n = 1,351	9.8% (62/635)	7.5% (54/716)
Rhodes et al. [40]	2002	United States	Inconsistent condom use during AI in the last year	MSM (overall), n = 498	34.0% (165/382)	41.4% (51/116)
Elford et al. [28]	2004	United Kingdom	UAI with serodiscordant or HIV-unknown male partner in the last 3 months	HIV-neg MSM, n = 1,254	26.9% (183/680)	18.6% (107/574)
			UAI with serodiscordant or HIV-unknown male partner in the last 3 months	HIV-pos MSM, n = 273	47.2% (67/142)	42.0% (55/131)
			UAI with male partner in the last 2 months	HIV-unk MSM, n = 570	35.6% (141/396)	19.0% (33/174)
Knapp et al. [29]	2004	United States	UAI in the last 2 months	MSM (overall), 551	23.8% (73/307)	19.7% (48/244)
Bolding et al. [31]	2005	United Kingdom	UAI with casual serodiscordant or HIV- unknown male partner in the last 3 months	HIV-neg MSM, n = 1,048	23.8% (75/315)	16.0% (117/733)
			UAI with casual serodiscordant or HIV- unknown male partner in the last 3 months	HIV-pos MSM, n = 661	43.3% (29/67)	18.0% (107/594)
			UAI with any casual male partner in the last 3 months	HIV-unk MSM, n = 291	20.3% (40/197)	5.3% (5/94)
Hospers et al. [26]	2005	Netherlands	UAI with casual partner in the last 6 months	MSM (overall), n = 6,185	23.0% (1,146/4,981)	21.0% (253/1,204)
Evans et al. [23]	2007	United Kingdom	UAI in the last 3 months	MSM (overall), n = 2,182	45.0% (929/2,065)	36.6% (43/117)
Xing et al. [7]	2008	Chinese	UAI with male partner in the last anal sex	MSM (overall), n = 269	58.1% (36/62)	34.8% (72/207)
Fernández-Dávila et al. [27]	2009	Spain	UAI in the last 3 months	MSM (overall), n = 2,044	31.0% (384/1,240)	27.0% (217/804)
Tsui et al. [1]	2010	Chinese	Engaged in any MSM UAI in the last year	MSM (overall), n = 566	53.1% (120/226)	33.8% (115/340)
Raymond et al. [41]	2010	United States	UAI	MSM (overall), n = 2,297	37.1% (268/455)	42.2% (664/910)
Liu et al. [39]	2011	Chinese	UAI in the last 3 months	MSM (overall), n = 2,692	59.9% (1,434/2,393)	64.9% (194/299)
Grov C. [42]	2012	United States	UAI in the last 3 months	MSM (overall), n = 477	28.2% (43/152)	20.9% (68/325)
Sanchez et al. [43]	2012	United States	UAI with most recent casual male sex partner in the last year	MSM (overall), n = 5,224	29.3% (766/2,617)	19.1% (497/2,607)

AI, anal intercourse; UAI, unprotected anal intercourse; *OR*, odds ratios; *CI*, confidence interval; HIV-neg, HIV-negative; HIV-pos, HIV-positive; HIV-unk, HIV-unknown.

### Overall results of the meta-analysis on the UAI prevalence

The summary of the prevalence of UAI among MSM is shown in Table 1. The results of the fourteen separate studies indicated that 33.9% (5,961/17,580) of MSM in the online group had UAI compared to 24.9% (2,700/10,853) of MSM in the offline group. As shown in Table 2, we found that the prevalence of UAI among online-based MSM was higher than the offline-based group in the overall analysis. The pooled *OR* was 1.35 (95% *CI* = 1.13-1.62, *P* < 0.01) for UAI between the online-based group and the offline-based group using the random effect model because between-study heterogeneity was significant (*Q* = 93.06, *P* < 0.001, *I*^*2*^ = 86.0%).

**Table 2:** Meta-analysis of the association between prevalence of UAI with MSM recruited online vs. offline

**Subgroups**	**Sample size**		**No. of studies**	**Test of association**^***#***^			**Test of heterogeneity**			**Egger’s test for publication bias**	
	**Online group**	**Offline group**		***OR (95% CI)***	***Z***	***P *value**	***Q***	***P *value**	***I***^***2 ***^**(%)**	***t***	***P *value**
Overall effects	17,580	10,853	14	1.35 (1.13, 1.62)	3.28	0.001	93.06	<0.001	86.0	0.16	0.880
European subsample	10,718	5,141	6	1.38 (1.17, 1.63)	3.84	<0.001	14.88	0.011	66.4	0.82	0.457
American subsample	4,181	4,866	5	1.21 (0.81, 1.82)	0.93	0.354	49.60	<0.001	91.9	−0.57	0.606
Asian subsample	2,681	846	3	1.63 (0.74, 3.62)	1.20	0.229	28.20	<0.001	92.9	1.37	0.402
Sample size > 500	16,984	10,205	11	1.32 (1.09, 1.61)	2.79	0.005	85.48	<0.001	88.3	-0.09	0.930
Sample size ≤ 500	596	648	3	1.51(0.89, 2.56)	1.52	0.128	7.40	0.025	73.0	2.62	0.232
UAI in the last 6 or more months	9,564	6,557	6	1.28 (0.94, 1.76)	1.56	0.12	62.11	<0.001	92.0	-0.79	0.850
UAI in the last 3 or less months	8,016	4,296	8	1.40 (1.13, 1.74)	3.03	0.002	30.44	<0.001	77.0	1.11	0.645

*OR*, odds ratios, *CI*, confidence interval, UAI, unprotected anal intercourse. ^*#*^The overall pooled estimate of risk OR obtained using DerSimonian and Laid method in the random effect model.

### Subgroup meta-analysis for UAI prevalence

We performed subgroup analyses stratified by the participants’ geographic location, the sample size and the time of last UAI in this study (Table 2). The subgroup analysis results showed that the prevalence of UAI was higher in the European subsample (*OR* = 1.38, 95% *CI* = 1.17-1.63, *P* < 0.01) and in sample sizes of more than 500 individuals (*OR* = 1.32, 95% *CI* = 1.09-1.61, *P* < 0.01) in the online group compared to the offline group. The prevalence of UAI was also significantly higher when the time of the last UAI was during the last 3 or more months (*OR* = 1.40, 95% *CI* = 1.13-1.74, *P* < 0.05) in the online group compared to the offline group.

### Sensitivity analysis

The effect of any single research study on the overall meta-analysis was carried out by deleting one study at a time. The exclusion of any individual study did not make a significant difference to this meta-analysis, suggesting that the results of our study are statistically reliable.

### Evaluation of publication bias

The Egger’s linear regression test was evaluated for funnel plot asymmetry (Table 3). The results showed that no publication bias existed in the overall analysis or the subgroup analysis.

**Table 3:** Egger’s linear regression test to measure the funnel plot asymmetric

**Subgroups**	**Y-axis intercept: α (95% *CI*)**
Overall effects	0.28 (-3.68 to 4.26)
European subsample	1.95 (-3.11 to 7.01)
American subsample	-2.08 (-10.59 to 6.43)
Asian subsample	8.09 (-29.29 to 45.47)
Sample size >500	-0.24 (-5.07 to 4.59)
Sample size ≤ 500	10.95 (-15.43 to 37.33)
UAI in last 6 or more months	-0.78 (-9.07 to 7.50)
UAI in last 3 months	1.11 (-3.36 to 5.58)

*CI*, confidence interval; UAI, unprotected anal intercourse.

## Discussion

We extracted fourteen studies that included 17,580 MSM who were recruited online (online group) and 10,853 MSM who were recruited offline (offline group) to evaluate the prevalence of UAI among MSM in this study. Our meta-analysis suggested that there was a higher prevalence of UAI among MSM who were recruited online than those recruited offline. The prevalence of UAI was defined differently in the fourteen studies of this meta-analysis, with the prevalence varying between 9.8% and 59.9% among MSM who were recruited online and between 7.5% and 64.9% among those who were recruited offline. These results suggest that the populations under study and the evaluation of risk groups vary in different, we should lead to caution when interpretating differences based on the mode of recruitment.

We performed subgroup analyses stratified by the participants’ geographic location (Europe, America and Asia), sample size (groups of more than 500 and less than or equal to 500 individuals) and the time of last UAI (UAI in the last six or more months and UAI in the last three or less months) to decrease the differences based on the mode of recruitment. The subgroup analyses showed that MSM who were recruited online were associated with increased UAI in studies with a sample size of more than 500 individuals but not in studies with a cohort of less than or equal to 500; this may have been due to reduced sampling bias in studies with larger samples. In addition, a high rate of UAI is primarily due to the definition or scope of UAI; therefore, if the definition or scope of UAI is broader (a broader definition of UAI means that the scope of UAI is unlimited or less limited), the UAI rate would be accordingly higher. Recently, a meta-analysis [15] demonstrated that online-based MSM were more likely to have UAI with male sex partners than offline-based MSM. That study provided the hypothesis for this meta-analysis and the results of that study [15] are consistent with our results, but that study did not perform subgroup analyses, such as subsample-based analyses, sample size-based analyses and last UAI time-based analyses.

Some limitations of this study should be discussed as well. First, only published studies were included in the present meta-analysis [44]. Thus, publication bias of our research may be possible; however, this was not observed in the statistical test. Second, this study assumes that the sample of MSM recruited online is representative of those MSM who met sexual partners online, and it also assumes that the sample of MSM recruited offline is representative of those MSM who met sexual partners at gay venues. Therefore, this study may have a generalization bias. In addition, statistically significance between-study heterogeneity was detected in the current study and may be distorting the results of this meta-analysis [45]. For example, men who were recruited through social networking sites, such as Facebook, are likely to be behaviorally very different from men who were recruited through sex-seeking sites, such as ManHunt. However, this was not a major problem because the self-reported risky sexual behavior involving UAI by MSM recruited online or offline was heterogeneous. Different subsamples may also contribute to the heterogeneity; therefore, the results of our meta-analysis should be interpreted with caution because the subsamples from the six countries used in this study were not uniform.

Our study found that MSM who were recruited online are more likely to engage in UAI with male partners than offline-based MSM, so data obtained from MSM using convenient Internet networks can provide potentially powerful tools for informing public health interventions. Most of the previous studies were conducted using offline-recruited (venue-based) sampling methods among MSM. Researchers used the results of those studies to design intervention measures targeting ordinary MSM, and these measures may not be suitable for online-based MSM who seek sexual partners [1]. The Internet provides researchers with valuable opportunities for conducting behavioral surveys among MSM because some MSM who are at a high risk of STDs or HIV infection may not participate in research when the investigation is conducted in the gay-specific venues [28]. The reasons why online MSM may be engaging in more UAI compared with offline MSM are as follows, those online-based MSM are less likely to be tested for HIV and more likely to have UAI with partners compared with offline-based MSM [46], and online-based HIV-negative MSM are more inclined to have UAI with potentially serodiscordant partners than offline-based MSM as well [47]. The risk of HIV infection should decrease after both an increased rate of condom use and standardized STD treatment for MSM [48]. There are valuable interventions for AIDS/STD prevention and treatment among MSM through the Internet, including promoting the use of condoms and encouraging the treatment of STDs.

Specific Internet related interventions may be helpful for online-based MSM, and it is crucial for HIV/STD control and prevention staff to pay more attention to this population to increase the awareness of self-protection and decrease the risk of HIV/STDs among online-based MSM.

## Conclusions

Increased sexual risk behavior has been linked to MSM who seek their partners online. This new situation will have extremely important implications for worldwide HIV/AIDS prevention, and increased attention should be paid to MSM because of this association. This study supports the results that a substantial percentage of MSM who were recruited online are more likely to engage in UAI than MSM who were recruited offline. Because we only used published papers in this study, we also need to pay close attention to the influences of unpublished studies, such as dissertations and papers presented at various conferences, which may help to confirm the results of this study. Targeted interventions of HIV prevention programs or services are recommended when designing preventive interventions to be delivered through the Internet.

## Abbreviations

HIV: Human immunodeficiency virus; MSM: Men who have sex with men; UAI: Unprotected anal intercourse; STD: Sexually transmitted diseases; CNKI: China National Knowledge Infrastructure.

## Competing interests

The authors declare that they have no competing interests.

## Authors’ contributions

JM, HJ and DZ participated in the design of the study and data collection. YZ performed the statistical analysis. YZ and ZS conceived of the study and participated in its design and coordination and helped to draft the manuscript. All authors read and approved the final manuscript.

## Pre-publication history

The pre-publication history for this paper can be accessed here:

http://www.biomedcentral.com/1471-2458/14/508/prepub
